# Differences in Bacterial Communities and Pathogen Indicators of Raw and Lagoon-Stabilized Farm Dairy Effluents

**DOI:** 10.3390/microorganisms12020305

**Published:** 2024-01-31

**Authors:** Gabriela Illarze, Amabelia del Pino, Pilar Irisarri

**Affiliations:** 1 Laboratorio de Microbiología, Departamento de Biología Vegetal, Facultad de Agronomía, Universidad de la República, Montevideo 12900, Uruguay; gillarze@fagro.edu.uy; 2 Departamento de Suelos y Aguas, Facultad de Agronomía, Universidad de la República, Montevideo 12900, Uruguay; amabelia@fagro.edu.uy

**Keywords:** facultative stabilization lagoon, 16S rRNA sequencing, pathogen indicators, physicochemical composition, environmental risk

## Abstract

One practice for handling farm dairy effluent (DE) comprises recycling them to the soil with the challenge of balancing the tradeoff associated with environmental pollution through nutrient and microorganism loading. This study investigated seasonal bacterial community composition, diversity, abundance, and pathogenic indicators in untreated (Raw) and lagoon-stabilized (Lagoon) DE. The correlation between bacterial profiles and DE physicochemical characteristics was also analyzed. Pathogen-indicator bacteria were studied by enumerating viable counts and the bacterial community structure by 16S rRNA gene sequence analysis. Lagoon storage effectively reduced total solids (64%), suspended solids (77%), organic carbon (40%), and total nitrogen (82%), along with total coliforms, *Escherichia coli*, and enterococci. However, this efficiency was compromised in winter. Lagoon and Raw sample bacterial communities presented different compositions, with several environmental variables correlating to microbial community differences. Lagoon-treated DE exhibited the most diverse bacterial community, dominated by Firmicutes (40%), Proteobacteria (30%), and Bacteroidota (7.6%), whereas raw DE was mainly composed of Firmicutes (76%). Regardless of the season, dominant genera included *Trichococcus*, *Romboutsia*, *Corynebacterium*, and *Paeniclostridium*. Overall, the study emphasizes the importance of lagoon treatment for DE stabilization, showcasing its role in altering bacterial community composition and mitigating environmental risks associated with pathogens and nutrients, particularly in summer.

## 1. Introduction

Farm dairy effluents (DE), the wastewater produced from washing down the dairy holding yards during and after milking, is generally managed in South America through direct application to pasture as a fertilizer (raw) or following partial treatment in stabilization lagoons [1]. The development of a circular economy led to the recycling of organic materials in the farm to complete nutrient and C cycles in the system [2]. Nevertheless, careful management is required to use the effluent nutrient and water resources effectively, avoiding environmental impacts. One matter of concern in using DE as liquid organic fertilizer is its potential microbial pollution, as manure is a reservoir for many microbial populations, including pathogens, which can cause contamination and harm public and animal health [3].

Dairy production intensification entails high volumes of effluents, which must be treated and stored for efficient use as fertilizer for crop and pasture production. There is a higher risk of pathogen transfer into the food chain when fresh manure is applied to the land than when waste is stored in lagoons, among other reasons to decrease the pathogen number [4]. Two-stage stabilization lagoons (anaerobic followed by facultative lagoon) are one of the most common effluent management systems due to their low operation and maintenance costs in treating dairy effluent to remove significant amounts of suspended solids and organic contaminants [5]. However, this treatment may be ineffective in removing N and P and decreasing the number of bacterial indicators [6], potentially allowing prolonged survival of fecal indicators and opportunities for frequent reinoculation from recurring inputs to effluent storage [7,8].

Generally, the surface application of DE (tanks or irrigation pumps) occurs consistently throughout the year [1], although one of the challenges of DE reutilization as a pasture N supplier is its variable composition, complicating the prediction of its agronomic value [9]. Compositional variations in DE are likely due to the time of milking, age and breed of the herd, feed quality, wash-water management, time relative to lactation, and seasonal variations [6,10]. To assess the benefits and potential threats in the land application of DE to the environment, it is imperative to quantify the temporal variation in its nutrients and contaminants [11].

More information is needed on the microbial community composition and diversity of untreated and lagoon-stabilized DE and its seasonal variability. Other studies have proposed that the impact and the survival rate of microbes from manure waste-based products on soil microbiomes largely depend on the type of treatment applied to manure [12]. Many studies focus on environmental risk assessment by studying antibiotic-resistant bacteria or their genes. Still, some studies propose that the microbial community of manure waste-based products carries beneficial microorganisms with potential use as biofertilizers [13,14]. Thus, it is essential to expand this knowledge to fully understand how the DE application to land impacts soil microbial dynamics.

There is scarce information regarding the bacterial composition, diversity, and abundance of South American DE, either raw or lagoon-treated. Irazoqui et al. [15] recently analyzed the microbial communities of two serial full-scale stabilization lagoon systems using whole genome shotgun sequencing. The functional analysis of the genomes revealed the central metabolic pathways and attributed them to several key organisms.

The knowledge of DE bacterial composition may help to elucidate ecological and environmental issues as a potential invasion of these microbes into the resident soil microbial community, especially since there is much concern about raw DE pathogenic contamination. This work aimed to assess the microbial community and pathogenic bacterial indicator differences between raw and lagoon-stabilized DE, and to determine the variability with the season and the physicochemical characteristics of DE. We hypothesize that (1) raw and lagoon DE harbor different bacterial communities and potential pathogenic risk, and (2) these bacterial communities and pathogenic indicators change in response to the seasonal variability and physicochemical characteristics of DE.

## 2. Materials and Methods

### 2.1. Dairy Effluent Collection and Farm Characteristics

DE was collected from the dairy farm of Centro Regional Sur, Faculty of Agronomy (Universidad de la República), with geographic coordinates 34°36′47.83” S and 56°12′54.00” W. Samples were collected on 5 September 2019, 7 November 2019 (spring–summer season), 11 May 2020 (autumn), and 18 August 2020 (winter), with mean seasonal water temperatures of 21.8, 26.4, 19.0, and 13.7 °C, respectively. Throughout the study, the farm ran a milking herd of 200 cows that grazed temperate pastures year-round.

The farm site’s effluent management system comprises a conventional two-stage dairy-shed waste-stabilization lagoon system (an anaerobic and a facultative lagoon operated in series) with a solid trap for effluent pretreatment. The system received the effluent generated by flushing the milking parlor and holding yard and chemically cleaning the milking machinery with sodium hypochlorite. The raw DE (Raw) was collected immediately after the cleaning from a holding tank (for solid trap) into a 2 L sterile container. The liquid portion undergoes gravimetric flushing into the two stabilization lagoons operated in series, with a hydraulic retention time of 119 days. The lagoon DE (Lagoon) was collected in a 2 L sterile container 15 cm below the surface from four different cardinal points at the facultative lagoon of 711 m^3^ volume and then composited. Three composite replicate samples were obtained from each type of DE and stored refrigerated at 4 °C (chemical analyses) or frozen at −20 °C (molecular analyses).

### 2.2. Characterization of Dairy Effluents

The pH and electric conductivity (EC) were measured in a mixture of effluent and deionized water (1:2.5 *v*/*v*) through potentiometry. Total solids (TS) and suspended solids (SS) were assessed gravimetrically following Standard Methods 2540 [16]. Total organic carbon (TOC) was measured via oxidation using potassium dichromate, following Mebius’ technique [17]. Total nitrogen (TN) was analyzed by the Kjeldahl method [18]. Ammoniacal N (NH_4_^+^-N) was extracted using a 2 M KCl solution and quantified by colorimetric analysis according to Rhine et al. [19]. By subtraction, organic N concentration (Norg) was deduced from total Kjeldahl and NH_4_^+^.

To determine elements P, K, Ca, Mg, and Na, samples were treated with HCl (20%) and calcined at 550 °C for 5 h. Total P was determined by the molybdate blue method with ascorbic acid [20]. Ca and Mg were determined by atomic absorption spectrometry, while K and Na were determined by atomic emission spectrometry [21].

### 2.3. Quantification of Pathogenic Bacterial Indicators

A 2 L subsample from each DE was transported on ice to the laboratory for immediate analysis. Total coliforms, *E. coli*, and enterococci numbers were determined by a ten-fold dilution series of 10 mL of each DE in 90 mL of 0.01 M phosphate buffer followed by agitation for 30 min in an orbital shaker at 200 rpm. The diluted samples were triplicated on 3M™ *E. coli*/Coliform Petrifilm™ or Chromocult Enterococci agar (Merck, Darmstadt, Germany). After incubation at 37 °C for 24 h, characteristic blue colonies were counted as *E. coli* and red ones as total coliforms (CFU/100 mL) or incubated at 41 °C for 24 h to enumerate enterococci.

### 2.4. Dairy Effluents DNA Extraction and 16S rRNA qPCR and Gene Amplicon Sequencing

An aliquot of 100 mL of each DE sample was centrifuged at 10,000× *g* for 10 min at room temperature to obtain pellets. Following the manufacturer’s instruction, total DNA was extracted using the PowerSoil^®^ DNA Isolation kit (Qiagen^®^, Hilden, Germany) from 0.25 g fresh weight (FW) of each centrifuged sample of the DE. The DNA concentration and purity were determined with NanoDrop^®^ 2000c UV–vis spectrophotometry (Thermo Scientific, Waltham, MA, USA). The extracted DNA samples were stored at −20 °C before analysis.

Quantitative real-time PCR (qPCR) of the copy number (abundance) of total bacteria was conducted with forward primer 515F (5′-GTGCCAGCMGCCGCGGTAA-3′) and the reverse primer 806R (5′-GGACTACHVGGGTWTCTAAT-3′) with 300 bp in length. An initial incubation of 5 min at 95 °C was followed by 30 cycles of 30 s at 95 °C, 45 s at the annealing temperature of 55 °C, and 50 s of extension at 72 °C.

The bacterial community composition was determined based on the variable V3-V4 region of the 16S rRNA gene sequencing with forward primer 341F (5′-CCTACGGGNGGCWGCAG-3′) and the reverse primer 805R (5′-GACTACHVGGGTATCTAATCC-3′). Amplicon library preparation was performed using the DNA extracted from each DE sample and then sequenced by the Illumina MiSeq platform (Illumina, San Diego, CA, USA) at Macrogen Inc. Company, Seoul, Republic of Korea. The generated data (accession number PRJNA1068640) was processed, demultiplexed, and quality-controlled using the DADA2 pipeline [22] with the “consensus” method to remove any remaining chimeric and low-quality sequences. The taxonomic identification (with 99% similarity) was performed using the SILVA database (v. 138) [23]. The data were then analyzed in R using the Microeco package [24].

### 2.5. Statistical Analysis

Data were analyzed in R version 4.1.0 (R Core Team, 2019). One-way analysis of variance (ANOVA) for DE physicochemical characteristics and microbial indicators was performed to evaluate the effect of DE type or sampling date (seasonal effect). To define the statistical significance of the mean, Fisher’s LSD post hoc test was performed with a 95% degree of confidence (*p* < 0.05). The Chao1 and the Shannon diversity indexes were used to investigate bacterial community richness and evenness. β-diversity comparisons were completed to evaluate communities using the Bray–Curtis index distance method. Visual differences among DE type and sampling date were determined using principal coordinate analysis (PCoA). Statistical differences in β-diversity among DE bacterial communities were tested with PERMANOVA (permutational multivariate analysis of variance) [25], and the PERMANOVA assumption of equal variance between groups was tested with PERMDISP (permutational analysis of multivariate dispersions) [26] by using the Vegan package in R [27].

The LEfSe (linear discriminate analysis effect size) algorithm identifies the most biologically informative features (for example: organisms, genes, or pathways) that consistently explain differences between microbial communities by emphasizing statistical significance, biological consistency, and effect relevance [28]. This study used the LEfSe algorithm to identify microbes that characterized the differences between DE types.

To determine the associations among bacterial genus relative abundance and the physicochemical parameters of DE, redundancy discriminant analysis (RDA) was performed by using the Vegan package in R [27].

## 3. Results

### 3.1. Farm Dairy Effluents Physicochemical Characterization

The physicochemical characteristics of the inlet DE (Raw) and the treated DE (Lagoon) in a two-lagoon storage system were evaluated through seasonal sampling (Table 1). Throughout the study period, there was considerable variation in the physicochemical parameters of the Raw relative to Lagoon DE samples (Table S1). Raw exhibited a pH ranging from 6 to 8.5, while Lagoon was consistently slightly alkaline, 7.8 on average. In the coldest seasons (May and August, for the South Hemisphere), Lagoon had significantly higher pH than Raw, whereas the reverse was observed in September; no significant difference was noted in November (Table 1). Electrical conductivity (EC) again exhibited greater variability in Raw, ranging from 1.8 to 6.9. The highest EC value for Lagoon, around 2.6, was recorded in November sampling. Significant differences in EC between the DE types were observed only in November and May.

Raw DE generally presented a higher percent of TS and SS compared to Lagoon DE, with significant variability across sampling dates (Table 1 and Table S1). The TS in Raw ranged from 0.3 to 2.3% and SS from 0.1 to 0.8%, whereas Lagoon DE levels were around 0.3% and 0.1%, respectively. Following stabilization in the lagoon, the TS and SS in the DE were substantially reduced, except for winter sampling.

Despite the seasonal variations, the lagoon-stored DE consistently demonstrated significantly lower TOC and TN than the raw DE. The reduction in TOC averaged around 40%, and for TN, it was around 82%, except for the August sampling, which accounted for less than 20% of TN removal. Accordingly, NH_4_^+^ content in winter sampling was notably higher in Lagoon than Raw; however, this trend was reversed in the other three sampling dates. Organic N fluctuated between 38% and 93% of the total N in Raw and 36 to 77% in Lagoon. The highest % organic N/Total N corresponded to the coldest sampling date (August).

P content in Raw was highly variable, ranging 16–19 mg/L in September and August to 84–101 mg/L in November and May, whereas the total P in Lagoon ranged between 24 and 38 mg/L. Significantly higher P content in Lagoon was observed in September and August, while the highest P content for Raw was accounted for in November and May. In September, November, and May, Raw had higher cation content than Lagoon, but again, in winter sampling, Lagoon accounted for the highest cation content (Table S1).

When considering the seasonal effect of the physicochemical characteristics of DE (Table S1), Raw DE generally exhibited its lowest nutrient content in winter sampling. The highest difference in TN and cation content for Raw was observed between the winter and spring sampling dates. Conversely, the TOC was higher in winter than in spring. Lagoon DE also had higher TOC in winter, significantly higher than for the rest of the sampling dates (Table S1), and TN was also significantly higher in winter. However, the cation content in Lagoon decreased in winter sampling relative to the other three sampling dates.

### 3.2. Quantification of Pathogenic Indicator Bacteria in Farm Dairy Effluents

**Figure 1:**
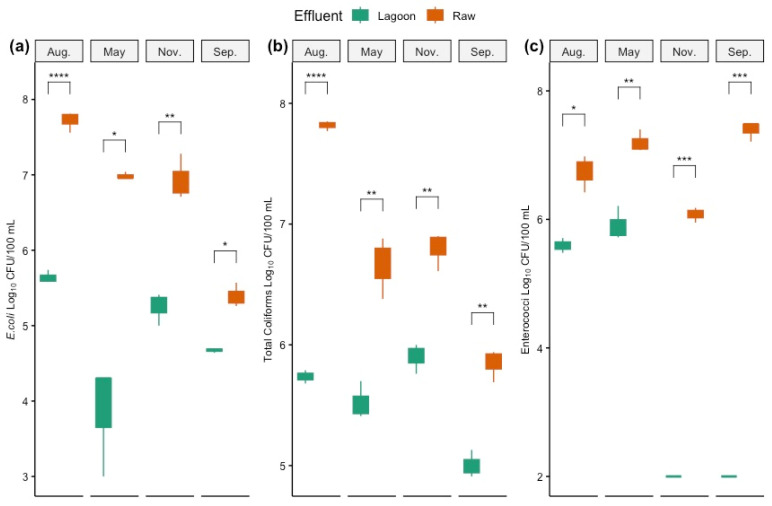
Numbers of *E. coli* (**a**), total coliforms (**b**), and enterococci (**c**) in the Raw and Lagoon dairy effluent samples (Log_10_ CFU/100 mL) at four sampling dates (September, November, August, and May) (*n* = 3 ± S.E). The length of each box represents the interquartile range IQR = Q 3 − Q 1; * *p* < 0.05, ** *p* < 0.01, *** *p* < 0.001, **** *p* < 0.0001.

The total coliforms, *E. coli*, and enterococci numbers were detected at higher levels in Raw than in Lagoon DE (Figure 1). The maximum difference in total coliform and *E. coli* was observed in August (winter), with 10^8^ CFU 100 mL^−1^ and 10^6^ CFU 100 mL^−1^ for Raw and Lagoon DE, respectively. Conversely, the maximum differences in enterococci were observed for September and November (spring–summer) with an average concentration of 10^7^ CFU 100 mL^−1^ and 10^2^ CFU 100 mL^−1^ for Raw and Lagoon DE, respectively.

Considering seasonal sampling, the highest counts of *E. coli* and total coliforms in Raw DE were recorded in winter (August sampling), and the lowest was accounted for in spring (September sampling). Similar patterns were observed for Lagoon DE, with *E. coli* and total coliforms significantly elevated in winter but not differing significantly from summer levels. Enterococci were near undetectable in Lagoon DE in spring–summer samplings (<10^2^ CFU 100 mL^−1^) but markedly increased in autumn. In contrast, enterococci count in Raw DE was the lowest in winter, and significantly higher counts were recorded in autumn (May sampling) and spring (*p* < 0.05).

### 3.3. Bacterial Abundance, Composition, and Diversity of Farm Dairy Effluents

**Figure 2:**
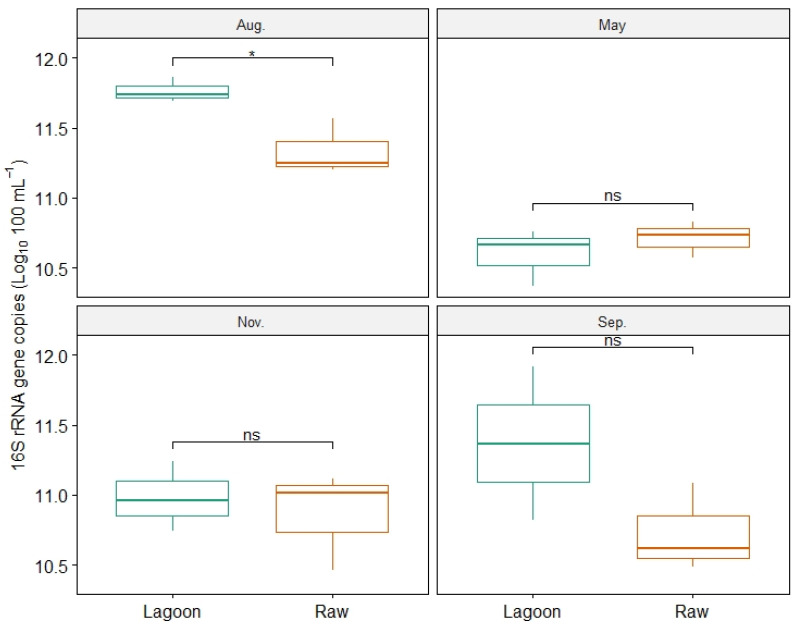
The gene copy numbers for bacterial 16S rRNA in the Raw and Lagoon dairy effluent samples at four sampling dates: August, May, November, and September. The length of each box represents the interquartile range IQR = Q 3 − Q 1, and the horizontal line is the median value; * *p* < 0.05, ns: not significantly different.

During the winter sampling, a higher abundance of bacterial copies was recorded for both DE types, and only at this sampling date were the bacterial 16S rRNA gene copy numbers significantly higher in Lagoon than in Raw DE (Figure 2). Bacterial copy numbers of Lagoon ranged from 4.3 × 10^10^ to 5.9 × 10^11^ copies 100 mL^−1^, whereas Raw DE ranged from 5.3 × 10^10^ to 2.3 × 10^11^ copies 100 mL^−1^.

Sequencing of the 16S rRNA V3-V4 region generated sequence reads from 24,582 to 39,412 with an average of 32,053 for Lagoon DE. For Raw DE, sequence reads varied from 13,461 to 40,616, averaging 27,451. In the Lagoon DE, the most abundant phylum was Firmicutes (40%), followed by Proteobacteria (30%) and Bacteroidota (7.6%), while in Raw, the phylum Firmicutes accounted for 76% of the total relative abundance, followed by Actinobacteriota (10%) (Figure S1).

**Figure 3:**
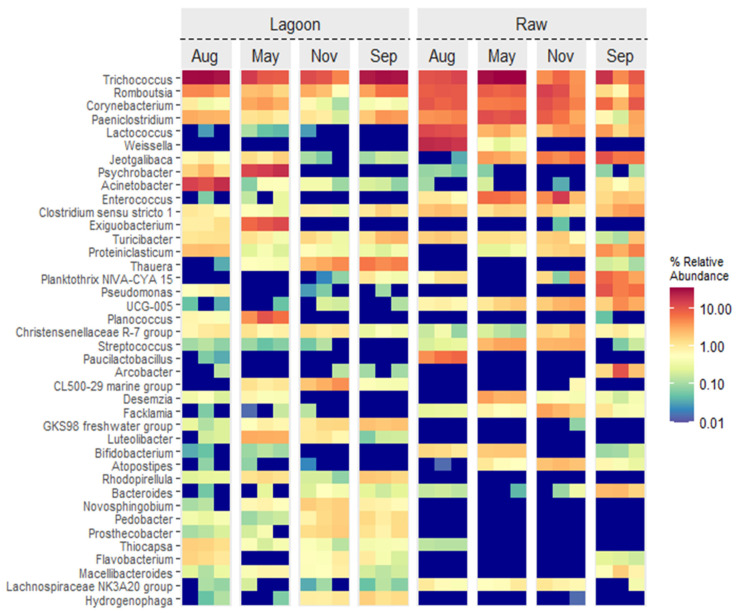
Microbial community heat map of the 40 most abundant genera in the Lagoon and Raw dairy effluent samples at four sampling dates: September and November (spring–summer), May (autumn), and August (winter).

A heat map of the top 40 genera in a total of 6392 ASVs is shown in Figure 3. Four genera comprised about 30% of the bacterial genes in the DE (Lagoon and Raw): *Trichococcus* (16–20%), *Romboutsia* (4–8%), *Corynebacterium* (1–8%), and *Paeniclostridium* (2–6%). Other abundant genera in Raw were *Lactococcus* and *Enterococcus*, accounting for 4 and 5%, respectively, while they were marginally detected in Lagoon samples. Certain genera exhibited noticeable seasonal variations in relative abundance. Specifically, *Jeotgalibaca*, *Psychrobacter*, *Exiguobacterium*, and *Planococcus* were more abundant in Lagoon during autumn (May) and winter (August) samplings than in spring–summer (September–November) samplings. In the Raw winter samples, however, *Weisella* and *Paucilactobacillus* had higher relative abundance, while *Jeotgalibaca* had lower relative abundance than the other sampling dates.

**Figure 4:**
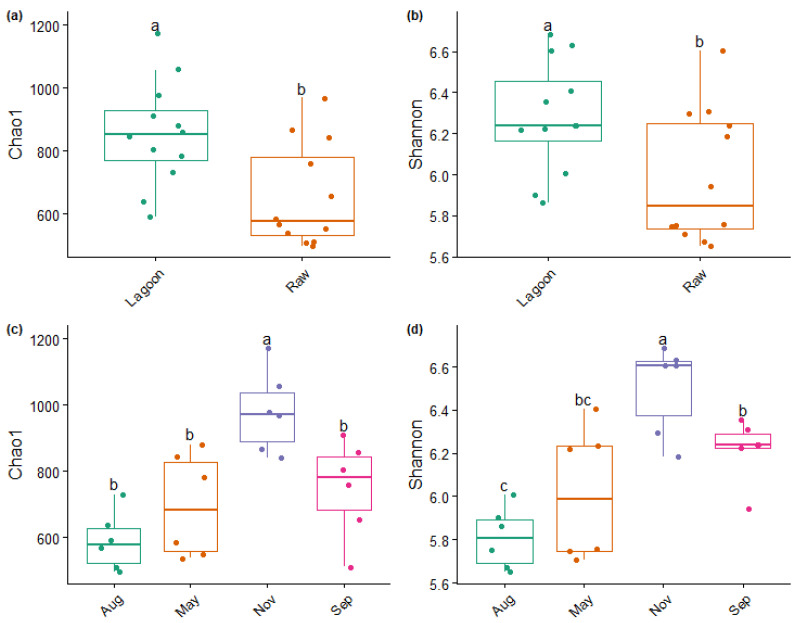
Bacterial richness (Chao1 index) and evenness (Shannon index) according to the type of farm dairy effluent (Raw or Lagoon) (**a**,**b**) or the sampling date (**c**,**d**); Aug: August, May, Nov: November, Sep: September. Different letter indicate significant differences at *p* < 0.05 between Raw and Lagoon dairy effluents or between sampling dates.

The PERMDISP analysis revealed no significant difference in dispersion from Raw and Lagoon and the sampling dates (*p* > 0.05). This suggests homogeneity of variance, indicating that differences in α- and β-diversities primarily resulted from dissimilarity rather than dispersion between DE type and sampling dates. Regardless of the sampling date, Lagoon DE had a significantly higher α-diversity than Raw DE (Figure 4a,b). Within each sampling date, November sampling (spring–summer) had significantly higher Chao1 and Shannon indexes than the other sampling dates (Figure 4c,d). In contrast, the August sampling had the lowest α-diversity indexes.

**Figure 5:**
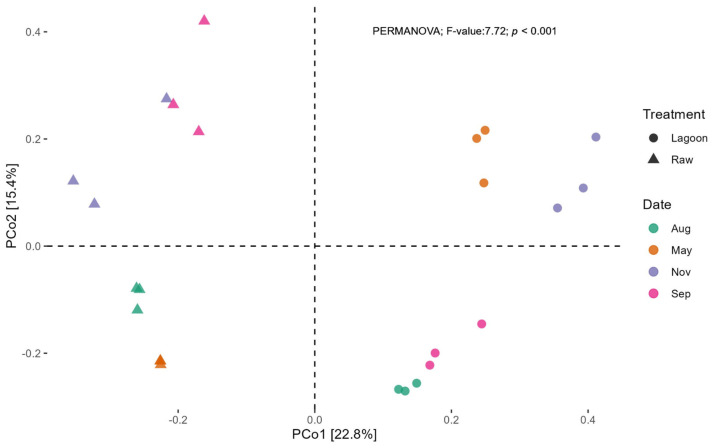
Principal coordinate analysis (PCoA) of bacterial communities based on the ASVs of the 16S rRNA gene sequencing from the Raw and Lagoon dairy effluent samples at each sampling date; Aug: August, May, Nov: November, Sep: September.

PCoA plots, using a Bray–Curtis distance matrix, also showed that bacterial communities grouped into two clusters (Figure 5, PERMANOVA R = 0.77, *p* < 0.05). The first two principal coordinates, PC1 and PC2, explained 22.8% and 15.4% of the data variation, respectively, clearly separating the communities of Raw from Lagoon. The separation of bacterial communities by sampling date was also visible in the PCoA (Figure 5). For Raw, the bacterial community was segregated into the spring–summer (September–November) and the autumn–winter (May–August) clusters. For Lagoon, however, the September sampling grouped with August, while the May and November sampling were distant from them and between each other.

**Figure 6:**
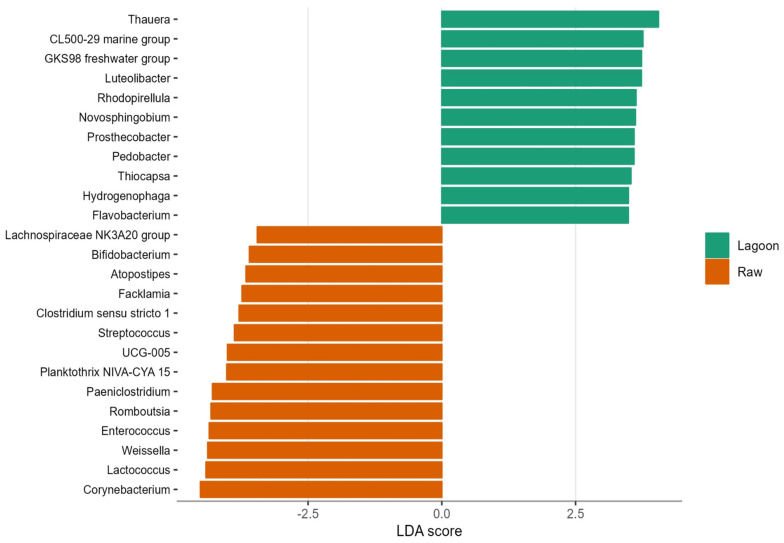
Histogram of linear discriminate analysis effect size (LEfSe) analysis of the 25 most abundant genera in the Lagoon and Raw dairy effluent samples. Longer histogram bars indicate a larger impact of certain taxa on the difference between the type of farm dairy effluent (*p* < 0.05).

The LEfSe analysis was carried out to identify the genera that predominantly contribute to explaining the greatest differences between bacterial communities of Lagoon and Raw DE (Figure 6). Among the 129 genus/ASVs that showed significant changes in their relative abundances, 13 were particularly representative of Raw DE, encompassing Firmicutes (*Weissella*, *Lactococcus*, *Romboutsia*, *Enterococcus*, *Paeniclostridium*, *Atopostipes*, *Facklamia*, *Streptococcus*, *Clostridium sensu stricto* 1, UCG-005), Actinobacteriota (*Corynebacterium* and *Bifidobacterium*), and Cyanobacteria (*Planktothrix* NIVA-CYA 15). Within Raw DE, *Corynebacterium*, *Lactococcus*, and *Weissella* emerged as the most significant genera (LDA score ≥ 4.4), whereas, Proteobacteria (*Thauera*, GKS98 freshwater group, *Novosphingobium*, *Thiocapsa*, *Hydrogenophaga*, *Ottowia*), Actinobacteriota (CL500-29 marine group), Verrucomicrobiota (*Luteolibacter*, *Prosthecobacter*), Planctomycetota (*Rhodopirellula*), and Bacteroidota (*Pedobacter*, *Flavobacterium*) had a greater abundance in Lagoon, with *Thauera* differentially predominant (LDA score > 4.0).

### 3.4. Linking Bacterial Community with Farm Dairy Effluent Characteristics

**Figure 7:**
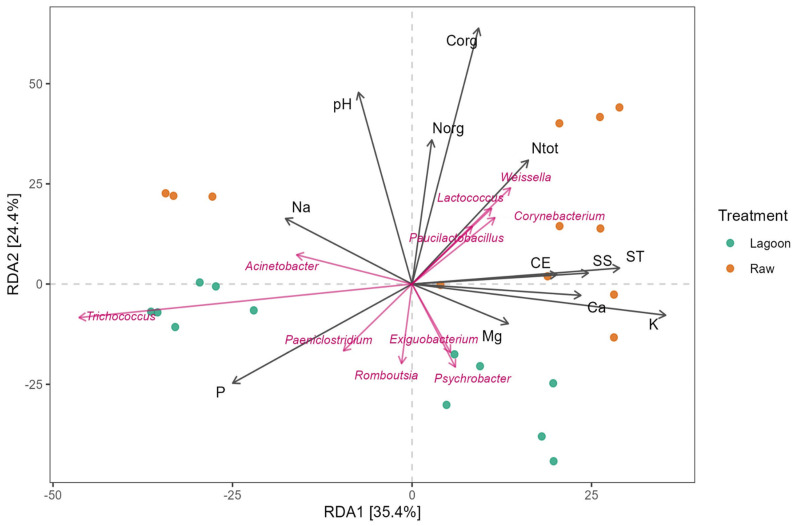
Relationship between bacterial communities and physicochemical parameters of farm dairy effluents (Raw or Lagoon) revealed by redundancy analysis (RDA). Pink arrows: dominant genera; black arrows: physicochemical properties of dairy effluents. Green and orange points correspond to the bacterial community of the Raw and Lagoon dairy effluent samples, respectively. Corg: total organic carbon, Ntot: total nitrogen, Norg: organic nitrogen, CE: electrical conductivity, ST: total solids, SS: suspended solids.

RDA was performed to elucidate the potential relationship between the bacterial community composition and the physicochemical properties of DE (Figure 7). The results showed that the first two axes explained 60% of the total variance. Both DE types are clustered separately, with the Raw DE generally exhibiting the highest values for the physicochemical parameters, as outlined in Table 1. Among the factors influencing microbial community composition, TOC emerged as the most significant, followed by pH, TP, K, and TN. The Mantel test supports these findings, revealing significant correlations with organic N, NH_4_, TS, SS, Na, Ca, and Mg (Table S2).

Genera, including *Weissella*, *Corynebacterium*, *Lactococcus*, and *Paucilactobacillus,* were mainly associated with TN. In contrast, *Psychrobacter*, *Exiguobacterium*, *Romboutsia*, and *Paeniclostridium* were negatively correlated with TOC, TN, organic N, and pH. In contrast, the *Paeniclostridium* was correlated with TP, and *Acinetobacter* was correlated with Na content.

## 4. Discussion

### 4.1. Bacterial Community Composition Changed with Farm Dairy Effluent Treatment and Season

The dairy industry generates large volumes of effluents that are applied to land as organic fertilizers throughout the year, but it is not without environmental concerns. The study of DE’s microbial composition, nutrient-value content, and seasonal variation is imperative to designing best management practices for appropriate DE disposal, and this matter needs to be thoroughly evaluated. This study compared the physicochemical and bacterial composition of untreated (Raw) from that of lagoon-stabilized DE (Lagoon) and their variation across seasonal sampling. Our work revealed the first insights into the changes in the microbial community structure of the Raw samples from that of the Lagoon samples. Notably, Lagoon DE exhibited a distinct bacterial community composition, as evidenced by the microbial ASVs, alpha diversity indices, and PCoA (Figure 3, Figure 4 and Figure 5). We found phyla Firmicutes and Proteobacteria almost proportionally dominant in Lagoon DE (40–30%), followed by Actinobacteriota and Bacteroidota (Figure S1). In contrast, Raw was overwhelmingly dominated by Firmicutes (76%). Those phyla were consistently dominant in DE, and the bacterial community was relatively stable at the phylum level; however, the composition of dominant genera varied considerably in different seasons (Figure 3).

Irazoqui et al. [15] performed taxonomic profiling of facultative lagoons from Argentina dairy industries, showing the dominance of phyla Proteobacteria and Actinobacteriota and, in less proportion, Firmicutes and Bacteroidota. Overall, there were no major differences in dominant phyla from our study’s bacterial community of the facultative lagoon, suggesting a core microbial selection by this treatment system (Figure S1). In addition, this observation aligns with the findings of Pandey et al. [29], suggesting that lagoon environments develop similar microbial profiles over time. Coinciding with our findings, Irazoqui et al. [15] also found members of *Patescibacteria*, *Verrucomicrobia*, and *Erysipelotrichales* to be in lower abundance in facultative lagoons (Figures S1 and S2).

Differential microbial community composition was also reported in a DE treatment plant [30,31]. These studies found that Firmicutes was the dominant phylum in raw (inlet) DE, but it decreased after lagoon treatment, while Proteobacteria increased in the anoxic–oxic zone. This difference may be explained by the growth inhibition of many of the obligate anaerobic members of the Firmicutes in aerobic conditions, as suggested by McGarvey et al. [31]. In accordance, Chang et al. [32] reported that treating DE with aeration and microalga and bacteria consortia led to the same shift in the dominance of Firmicutes to Proteobacteria. These findings coincided with our results, suggesting that Proteobacteria is also enriched during treatment in the facultative lagoon environment. Indeed, genera *Thauera*, GKS98 freshwater group, *Novosphingobium*, *Thiocapsa*, *Hydrogenophaga*, and *Ottowia* within Proteobacteria had the largest effect in differentiating the lagoon-treated DE from the untreated one (Figure 6). These genera have been isolated from several sludge-based anaerobic wastewater treatment plants and identified as key members for removing N [30,33,34].

In contrast, the genera *Corynebacterium*, *Enterococcus*, *Lactococcus*, and *Weissella* were associated with raw DE (Figure 3 and Figure 6), most of which can be found in raw milk and cattle feces [35,36], which were regular inputs present in fresh effluents.

Among the genera detected, *Trichococcus*, *Romboutsia*, *Clostridium senso stricto* 1, and *Paeniclostridium* were highly abundant in both raw and lagoon-treated DE and at all sampling dates. They were also major genera frequently detected in dairy manure treatments [29,30,31,37,38]. Most Firmicutes have higher environmental adaptability and can degrade various complex organic materials [39]. Furthermore, members of Firmicutes and Bacteroidota have been reported to be present in different reactors because of their ability to degrade organic compounds into monomers [40]. In this sense, Irazoqui et al. [15] identified *Bacteroidetes* and *Clostridiales* as key members of the communities in facultative ponds involved in polymer and amino acid metabolism, respectively. They suggest that the high amount of carbohydrates and proteins dumped in these ponds would make *Clostridiales* important community members. Other studies based on metagenome-assembled genomes indicate that *Clostridiales* also could play a role in polysaccharide degradation with substrates rich in cellulose [41]. A previous study also reported that biogas slurry application enriched the genera *Paeniclostridium* and *Romboutsia* in the soil, and they were implicated in the transmission of antibiotic resistance genes (ARGs) [42].

Additionally, some archaea families were identified in both DE in less than 0.1% relative abundance . *Methanosaetaceae*, a family of archaeal acetoclastic methanogens, occurs in Lagoon DE. This is the predominant acetoclastic methanogen in anaerobic digestor systems [43]. *Methanosphaera* and *Methanobacteriaceae,* archaeal families of hydrogenotrophic methane producers, were detected in Raw DE. *Methanobrevibacter*, another hydrogenotrophic or formate methane producer, was present in both the Raw and Lagoon DE samples. Identifying these archaeal families is of particular importance, given the interest of the dairy industry in performing anaerobic digestion for energy production [44].

This study observed seasonal changes in the composition of the bacterial community (Figure 3, Figure 4 and Figure 5). The bacterial diversity indices increased in the spring/summer sampling (November, Figure 4). This increase might be attributed to the higher temperatures that produce the fastest-growing microorganisms that accelerate organic matter decomposition. Although our study accounted for the higher total bacterial abundance in the winter sampling for both DE (Figure 2), another study also found the lowest bacterial richness and diversity to be in winter in a wastewater treatment plant [45], and higher concentrations of microorganisms were reported in summer than in the winter in air samples collected near the dairy sludge tanks [46].

Although the highly abundant bacterial communities did not vary with the sampling date, some genera showed a remarkable shift between the warm and cold sampling dates. For example, the genera *Jeotgalibaca*, *Psychrobacter*, *Exiguobacterium*, and *Planococcus* were enriched in the Lagoon samples during autumn and winter, *Acinetobacter* were enriched in autumn, while *Weisella* were enriched in the Raw samples (Figure 3). In contrast, *Thauera* had a greater relative abundance in the warmest samplings in Lagoon, while *Jeotgalibaca* decreased in Raw during the winter (Figure 3). Other studies have reported this seasonality in the microbial community of different DE and wastewater treatment systems and attributed these changes mostly to environmental temperatures [38,45,47].

### 4.2. Variability in Physicochemical Properties of Farm Dairy Effluents and Their Relation with Bacterial Community Structure

The variation in physicochemical parameters has both direct and indirect influence on the activities of microorganisms. In this study, the Mantel test and RDA analysis (Figure 7) revealed that the community composition of bacteria is significantly affected by different physicochemical parameters of DE. The main factors influencing the bacterial community were TOC, pH, and TN content. The dominant heterotrophic bacteria belonging to Firmicutes and Proteobacteria may be implied in the organic C and N turnover in the DE. Indeed, as indicated in Figure 7, the highly abundant genera *Weissella*, *Corynebacterium*, *Lactococcus*, and *Paucilactobacillus* correlated significantly with TN, indicating that they play key roles in transforming N compounds. TN, K, Na, pH, and suspended solids have been previously demonstrated to correlate with bacterial community structure [48,49].

The RDA analysis also revealed that the stabilization lagoon system had lower levels of organic carbon (TOC), nitrogen (TN and organic N), solids (ST and SS), and EC (Figure 7), accounting for the expected lagoon purposes. However, there was high variability in nutrient removal efficiency along the seasonal sampling. For example, a lower efficiency of TN removal (20%) at treating DE in winter was observed in the Lagoon samples (Table S1). Therefore, considering that low temperatures decrease the activity of various microorganisms and slow the rate of organic matter degradation, it was expected that the TOC in DE would be higher in winter sampling and, accordingly, higher TN and lower cation content concentrations were found in the Lagoon samples (Table 1 and Table S1). Previous studies showed similar results [38,50]. *Thauera* species were as identified as major denitrifying bacteria that occurred in digested DE [51], and their increase revealed in the Lagoon samples during the warmest sampling may suggest a role in N removal, as postulated by Ren et al. [34].

### 4.3. Risk of Pathogen Loads from Farm Dairy Effluent

Effective management of pathogenic risks linked to DE treatment is critical for public health. Treated effluents from lagoon storage systems may still contain pathogenic bacteria, posing a substantial risk of transporting zoonotic pathogens from land-applied DE through runoff [52]. Our study delves into the risks posed by untreated DE and the efficacy of lagoon stabilization in reducing pathogen loads across varying seasons. This study used pathogenic indicator bacteria such as *E. coli*, total coliforms, and enterococci as biomarkers to infer their reduction through DE treatment. The results demonstrated an overall significant effect of the lagoon stabilization of DE on the microbial concentration of indicator microorganisms. As expected, the number of bacterial biomarkers was highest in the raw DE. After lagoon stabilization treatment of the DE, *E. coli* and total coliform numbers declined almost two orders of magnitude (Figure 1), but were still too high to achieve the levels recommended by the National Council for discharging to waterways (5.0 × 10^3^ UFC/100 mL) [53].

The sequencing analysis of 16S rRNA revealed that raw DE had dominant genera such as *Enterococcus*, *Streptococcus*, and other possible pathogenic bacteria such as *Facklamia* (Figure 3) [54], all of which are manure-borne microorganisms regularly detected in cattle manures and wastewaters [4,55]. The lagoon treatment could effectively eliminate the majority of pathogenic bacteria, as the occurrence of these genera was not detected or was in very low abundance. Most dominant pathogenic bacteria, such as *Escherichia coli* O157:H7, *Salmonella*, *Listeria*, and *Campylobacter* [56,57,58], etc., associated with cow manure and DE, may contaminate the forage crops when applied [4]. In our study, these genera were not identified in either type of DE).

The season had a crucial effect on the persistence of pathogenic indicators [59]. The fact that the highest counts of *E. coli* and total coliforms were recorded in winter for both types of DE indicates greater consideration should be given to its environmental impact during winter application. Indeed, it has been shown that the mechanism of pathogen removal in natural attenuation lagoons has been associated with effluent exposure to sunlight as a natural source of bacterial inactivation by UV light [60], explaining the higher performance of lagoons in summer to reduce the number of pathogen indicators. A study conducted in covered and open swine lagoons also showed that the reduction in solar radiation contributed to the survival of bacterial indicators [48]. However, contrary to our findings, they reported higher counts of pathogen indicators in swine lagoons occurring in summer and the lowest in winter, but the densities of these bacteria were more associated with the physicochemical composition of the wastewater rather than the season.

## 5. Conclusions

Our results highlight the lagoon system’s effectiveness in altering bacterial community composition and reducing pathogen indicator levels, particularly in summer.

The 16S rRNA gene sequencing analysis demonstrated that a large, diverse bacterial population inhabits the lagoon-stabilized DE and changes with the season.

These results emphasize the importance of adopting lagoon systems as a good practice for mitigating the risks associated with pathogen loads from DE. However, the observed decline in treatment efficiency during winter warrants further investigation to enhance the robustness of lagoon systems under diverse environmental conditions. Knowledge concerning the DE microbial community composition and its functional potential is crucial to improving the efficiency and safeness of DE stabilization and application to pastures.

## Figures and Tables

**Table 1:** Physicochemical characteristics of the farm dairy effluents obtained immediately after washing off the milking parlor (Raw) or from the lagoon storage system (Lagoon) at four seasonal sampling dates (*n* = 3 ± S.E).

						mg L^−1^							
Month	Effluent	pH	EC (mS m^−1^)	TS (%)	SS (%)	Corg	TKN	NH_4_^+^-N	P	K^+^	Na^+^	Mg^2+^	Ca^2+^
September	Raw	8.5 ± 0.0 a	2.4 ± 0.0 a	0.8 ± 0.4 a	0.8 ± 0.4 a	514 ± 80 a	800 ± 39 a	216 ± 23 a	16 ± 1 b	722 ± 88 a	217 ± 17 a	100 ± 26 a	197 ± 61 a
	Lagoon	7.7 ± 0.0 b	2.1 ± 0.1 a	0.1 ± 0.0 b	0.1 ± 0.0 b	359 ± 12 b	137 ± 14 b	87 ± 15 b	38 ± 1 a	150 ± 18 b	144 ± 17 b	31 ± 4 b	48 ± 5 b
November	Raw	8.2 ± 0.4 a	6.9 ± 1.2 a	0.3 ± 1 a	0.3 ± 1 a	643 ± 35 a	538 ± 11 a	332 ± 47 a	84 ± 19 a	436 ± 107 a	131 ± 28 a	46 ± 9 a	76 ± 15 a
	Lagoon	7.7 ± 0.0 a	2.3 ± 0.1 b	0.1 ± 0.0 b	0.1 ± 0.0 b	414 ± 19 b	95 ± 7 b	39 ± 1 b	36 ± 3 b	156 ± 13 b	75 ± 8 b	26 ± 3 b	47 ± 5 b
May	Raw	7.3 ± 0.0 b	4.8 ± 0.1 a	0.6 ± 0.1 a	0.6 ± 0.1 a	736 ± 145 a	539 ± 9 a	75 ± 10 a	101 ± 5 a	277 ± 21 a	226 ± 19 a	73 ± 5 a	87 ± 5 a
	Lagoon	7.8 ± 0.1 a	3.8 ± 0.0 b	0.1 ± 0.0 b	0.1 ± 0.0 b	275 ± 18 b	88 ± 2 b	37 ± 1 b	24 ± 4 b	224 ± 27 a	157 ± 15 b	38 ± 6 b	32 ± 6 b
August	Raw	6.0 ± 0.0 b	1.8 ± 0.0 a	0.1 ± 0.0 a	0.1 ± 0.0 a	890 ± 22 a	281 ± 17 a	19 ± 1 b	19 ± 2 b	69 ± 4 b	16 ± 1 b	8.3 ± 1 b	15 ± 1 b
	Lagoon	8.1 ± 0.0 a	2.3 ± 0.4 a	0.2 ± 0.0 a	0.2 ± 0.0 a	573 ± 42 b	231 ± 5 b	53 ± 4 a	26 ± 2 a	92 ± 12 a	52 ± 7 a	17 ± 2 a	23 ± 3 a

Different letters for each parameter indicate significant differences at *p* < 0.05 between Raw and Lagoon dairy effluents at each sampling date.

## Data Availability

Data are contained within the article and supplementary materials.

## Supplementary Materials

The following supporting information can be downloaded at: https://www.mdpi.com/article/10.3390/microorganisms12020305/s1, Figure S1: Relative abundances of the main bacterial phyla in the Lagoon and Raw dairy effluent samples at four sampling dates: August, May, November, September; Figure S2: Relative abundances of the main bacterial families in the Lagoon and Raw dairy effluent samples at four sampling dates: August, May, November, September; Table S1: Seasonal variation in physicochemical characteristics of the farm dairy effluents, obtained immediately after washing off the milking parlor (Raw) or from the lagoon storage system (Lagoon) (*n* = 3 ± S.E); Table S2: Relationships between bacterial community compositions at genus level and physicochemical properties of farm dairy effluents revealed by the Mantel test.
