# Supplementary material for: Differences in Bacterial Communities and Pathogen Indicators of Raw and Lagoon-Stabilized Farm Dairy Effluents
Source: Microorganisms. 2024 Jan 31;12(2):305. doi: 10.3390/microorganisms12020305 (PMC10893489; doi:10.3390/microorganisms12020305)
Supplement: Supplementary file 1 [file microorganisms-12-00305-s001.zip › microorganisms-2823584-supplementary.pdf]

Supplementary data

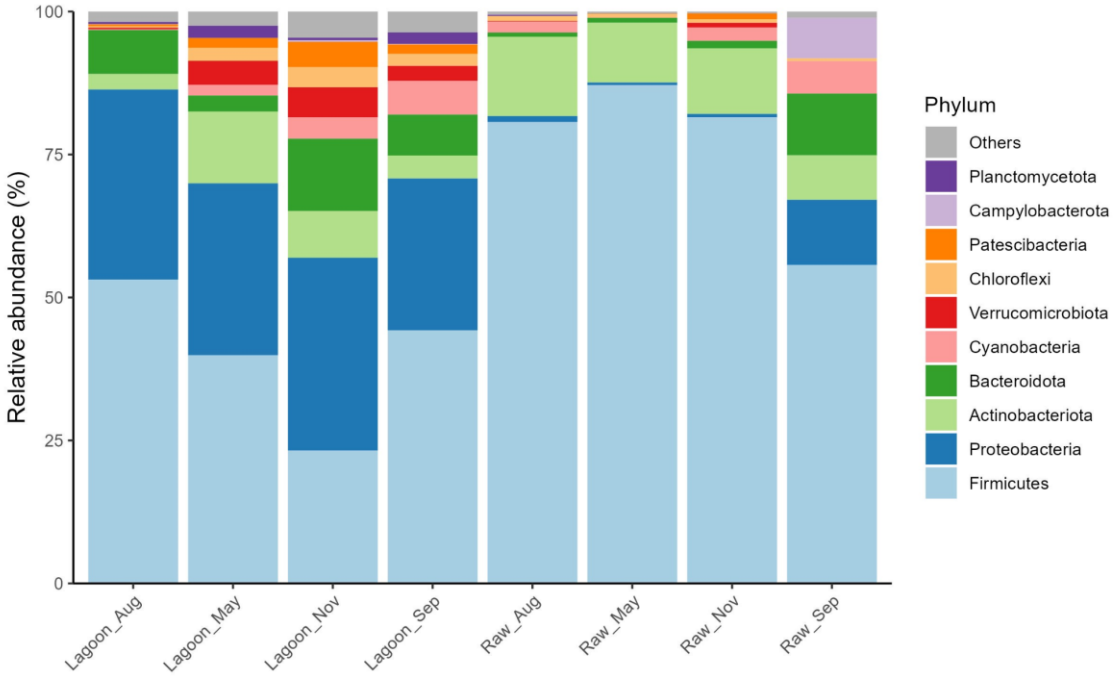

Figure S1. Relative abundances of the main bacterial phyla in Lagoon and Raw dairy effluents at four sampling dates: August, May, November, September.

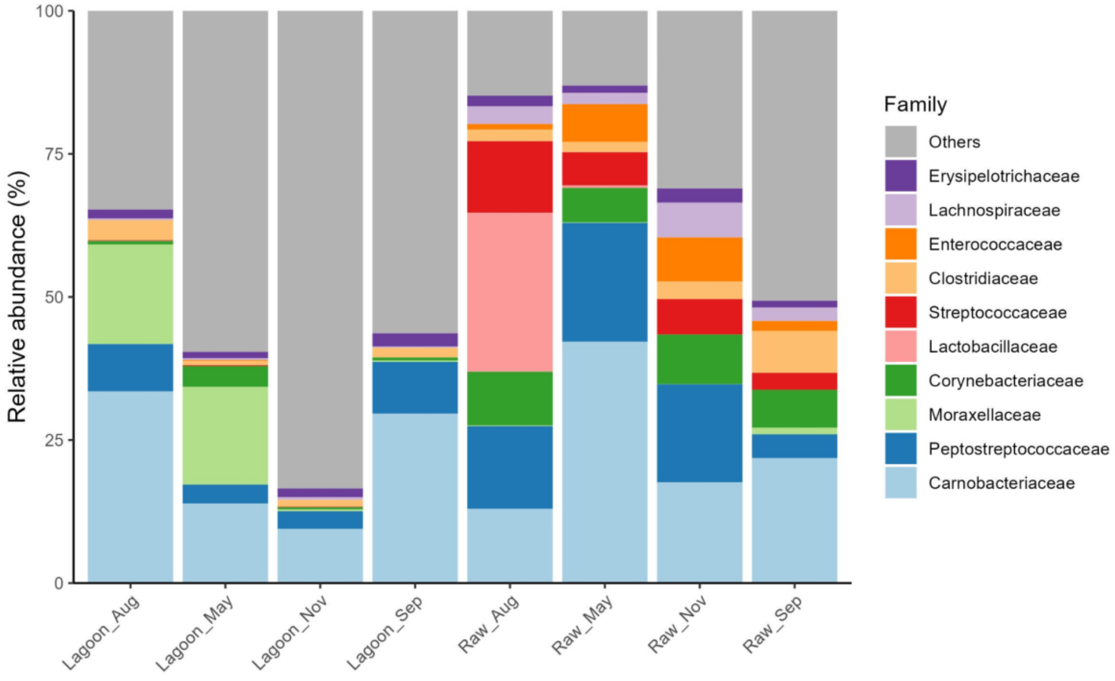

Figure S2. Relative abundances of the main bacterial families in Lagoon and Raw dairy effluents at four sampling dates: August, May, November, September.

Table S1. Seasonal variation in physicochemical characteristics of the Farm Dairy Effluents, obtained immediately after washing off the milking parlor (Raw) or from the lagoon storage system (Lagoon) (n=3± S.E).

						(mg L^{-1})							
Months	Effluent	pH	CE (mS m^{-1})	TS (%)	SS (%)	Corg	TKN	NH_4^+-N	P	K^+	Na^+	Mg^{2+}	Ca^{2+}
September	Raw	8.5±0.0 a	2.4±0.0 c	2.3±0.5a	0.8±0.4a	514±80b	800±39a	216±23b	16±1b	722±88a	217±17a	100±26a	197±61a
November		8.2±0.4 a	6.9±1.2 a	0.8±0.0b	0.3±1b	643±35ab	538±11b	332±47a	84±19a	436±107b	131±28b	46±9c	76±15b
May		7.3±0.0 b	4.8±0.1 b	0.9±0.0b	0.6±0.1b	736±145ab	539±9b	75±10c	101±5a	277±21c	226±19a	73±5b	87±5b
August		6.0±0.0 c	1.8±0.0 c	0.3±0.0b	0.1±0.0b	890±22a	281±17c	19±1d	19±2b	69±4d	16±1c	8.3±1d	15±1c
September	Lagoon	7.7±0.0 b	2.1±0.1 b	0.3±0.0a	0.1±0.0b	359±12c	137±14b	87±15a	38±1a	150±18b	144±17a	31±4ab	48±5a
November		7.7±0.0 b	2.3±0.1 b	0.3±0.0a	0.1±0.0ab	414±19b	95±7c	39±1bc	36±3a	156±13b	75±8b	26±3b	47±5a
May		7.8±0.1 b	3.8±0.0 a	0.3±0.0a	0.1±0.0ab	275±18d	88±2c	37±1c	24±4b	224±27a	157±15a	38±6a	32±6b
August		8.1±0.0 a	2.3±0.4 b	0.2±0.0b	0.2±0.0a	573±42a	231±5a	53±4b	26±2b	92±12c	52±7b	17±2c	23±3b

Table S2. Relationships between bacterial community compositions at genus level and physicochemical properties of farm dairy effluents revealed by Mantel test.

Variable	Correlation coefficient	p
pH	0.229	0.013
CE	0.152	0.106
ST	0.344	0.004
SS	0.285	0.010
Corg	0.299	0.003
Ntot	0.508	0.003
Norg	0.367	0.003
NH4	0.323	0.003
K	0.423	0.003
Na	0.181	0.019
Mg	0.293	0.005
Ca	0.352	0.004
P	0.056	0.223

$p < 0.05$ indicates significant correlation
